# Supplementary material for: Design and validation of a frugal, automated, solid-phase peptide synthesizer
Source: PLoS One. 2020 Aug 19;15(8):e0237473. doi: 10.1371/journal.pone.0237473 (PMC7437905; doi:10.1371/journal.pone.0237473)
Supplement: S1 File — Details in the respective sections on (A) wiring of the synthesizer, (B) sources and prices of reagents, (C) modification of the synthesizer to handle a greater number of amino acids, (D) the python script for operation of the synthesizer, (E) cost estimate of peptide product, and (F) testing of the immobilized HA-tag peptide. (PDF) [file pone.0237473.s001.pdf]

Supplemental Information for:

Design and Validation of a Frugal, Automated, Solid-Phase Peptide Synthesizer

Nathaniel E. Kallmyer, Nathan E. Rider, Nigel F. Reuel*

Iowa State University, Dept of Chemical and Biological Engineering

*Corresponding Author – reuel@iastate.edu

Contents

Supplement A. Wiring Diagrams 2

Supplement B. Sources and Prices of Reagents 2

Supplement C. Modification for a Greater Number of Inlets 4

Supplement D. Python3 Script for Synthesizer Operation 7

Supplement E. Reagent Cost Estimate 12

Supplement F. Testing HA-tag peptide 12

Supplement A. Wiring Diagrams

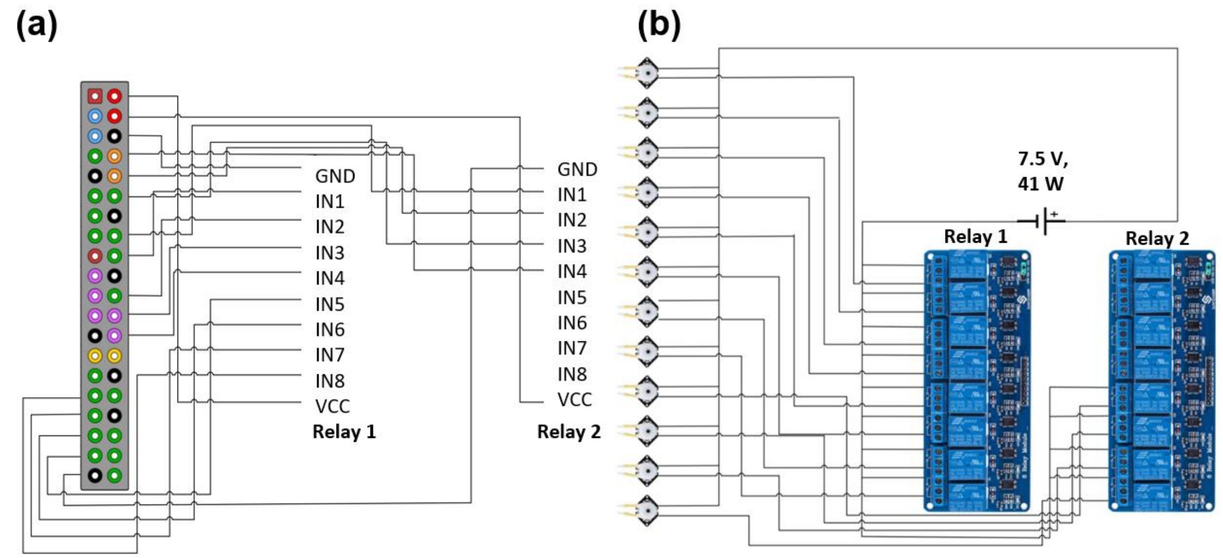

Fig A. Wiring diagrams of device. (**a**) Connection of Raspberry Pi 3B GPIO pins to relays. (**b**) Connection of DC power supply to relays and pump motors.

Supplement B. Sources and Prices of Reagents

Table A. Sources and prices of common SPPS reagents

Reagent	Supplier	Quantity	Price (USD) *Includes Hazmat packaging charge
NMM	Millipore Sigma	250 mL	22.70
DMF	Chem-Impex	1 L	43.50*
HATU	Chem-Impex	5 g	36.00*
Piperidine	Chem-Impex	1 L	108.50*
Fmoc-glycine-2-chlorotrityl resin	Chem-Impex	1 g	20.00
N^{ε}-Boc-L-lysine-2-chlorotrityl resin	Chem-Impex	1 g	20.00
Aminomethyl polystyrene resin	Chem-Impex	5 g	20.00

Table B. Quantities and prices of protected amino acids from ChemImpex

Reagent	Quantity	Price (USD)
Na-Fmoc-Nw-(2,2,4,6,7-pentamethyldihydrobenzofuran-5-sulfonyl)-L-arginine	5 g	25.00

Fmoc-L-aspartic acid b-tert-butyl ester	5 g	12.50
Fmoc-L-valine	5 g	10.00
Fmoc-L-alanine	5 g	10.00
Na-Fmoc-Nin-Boc-L-tryptophan	5 g	15.00
Fmoc-O-tert-butyl-L-serine	5 g	15.00
Na-Fmoc-Nim-trityl-L-histidine	5 g	15.00
Fmoc-L-proline	5 g	10.00
Na-Fmoc- Nd-trityl-L-glutamine	5 g	15.00
Fmoc-L-phenylalanine	5 g	10.00
Na-Fmoc-Ne-Boc-L-lysine	5 g	10.00
Fmoc-L-glutamic acid g-tert butyl ester hydrate	5 g	15.00
Fmoc-L-leucine	5 g	10.00
Fmoc-O-tert-butyl-L-tyrosine	5 g	20.70
Na-Fmoc-Nd-trityl-L-glutamine	5 g	15.00
Fmoc-glycine	25 g	12.50
Na-Fmoc-Ng-trityl-L-asparagine	5 g	12.50

Supplement C. Modification for a Greater Number of Inlets

The demonstrated peptide synthesizer was functional but only capable of handling 6 amino acids at any single time. Peptides with more than 6 different residues required manual changing of feed lines between synthesis steps. To fully automate synthesis steps, it would be necessary to control a greater number of pumps. Because the Raspberry Pi (v3) only features 27 GPIO pins, signal multiplexing may be necessary to achieve the necessary number of outputs. By coupling the GPIO pins with binary decoders (Fig B), it is possible to greatly increase the Raspberry Pi's capacity for pump control. Rather than have separate GPIO pins control individual pumps, binary decoders assign a binary permutation to a pump. In the below proposed design, 3 4-bit decoders are used to direct power to a maximum of 45 different pumps. 4 binary GPIO outputs are used to direct the output of the decoder (A0-A3), and 3 binary GPIO outputs are used to enable and select which chip outputs signal. A decoder chip may be toggled "on" by sending a low "enable" (E) signal. The first output of each chip (Y0, corresponding to a 0000 input) is left disconnected to allow for an "off" state when no signal is supplied to the chips. Sample inputs and outputs are provided in Table C. While the three enable inputs do allow for simultaneous control of up to three pumps, this is not an intended function and may result in overcurrent. It is important to be sure that the decoder employed has output suitable for the employed relay. If an "active low" (switch turns "on" when low signal is received) relay is employed, an active low decoder must also be used. Similarly, "active high" relays will require active high decoders. This is necessary to confine control to a single pump at any time. Single pole double throw (SPDT) switches may tolerate either form of input if pumps are connected to the correct output line.

The below design was prepared to minimize complexity rather than optimize efficiency. Inverters may be added to the "enable" (E) inputs to provide a default "off" state, allowing assignment of the low signal output (Y0) to pumps. A higher-bit decoder (6-bit) may also be used; however, this may require mounted on a printed circuit board. Mountable GPIO extenders or "hats" may also be added to expand GPIO output; however, these may require software installation and daisy chaining to achieve the desired number of outputs.

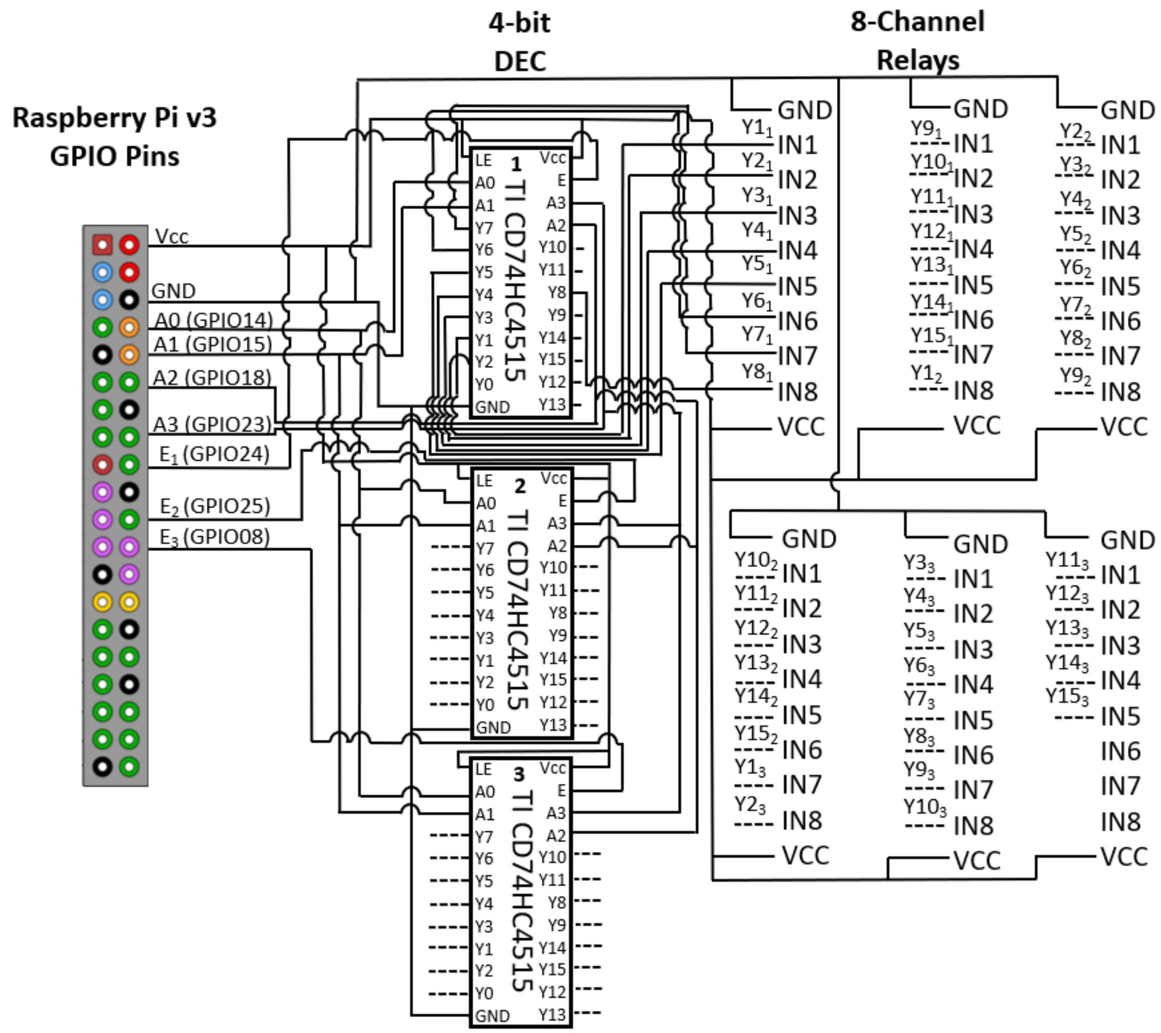

Fig B. Wiring diagram of GPIO outputs to control a greater number (40+) of pumps. Additional pull-down resistors may be necessary for each GPIO input to prevent floating conditions.

Table C. GPIO Multiplexing Truth Table

A0	A1	A2	A3	E_1	E_2	E_3	Output
0	0	0	0	0	0	0	$Y0_1$, $Y0_2$, $Y0_3$ (off)
0	0	0	0	0	1	1	$Y0_1$
1	0	0	0	0	1	1	$Y1_1$
0	1	0	0	0	1	1	$Y2_1$
0	0	1	0	0	1	1	$Y4_1$
0	0	0	1	0	1	1	$Y8_1$
1	1	0	0	0	1	1	$Y3_1$
1	1	0	0	1	0	1	$Y3_2$
1	1	0	0	1	1	0	$Y3_3$
1	1	0	0	0	0	1	$Y3_1$, $Y3_2$ (multiple “on” outputs, undesired)

Use of this design will require modification to the provided Python script. While the original script assigns a single GPIO output to a pump channel, this new script would require a specific combination of GPIO outputs. Rather than assign a single on/off state to a single GPIO pin, an array of on/off states must be assigned to correspond to a relay number. In the absence of pump operation, all enable outputs should be set to "GPIO.HIGH" or "1" to prevent pump operation prior to complete entry of the on/off combination. These instructions will assume that GPIO channels are assigned in the order of A0-A3 then E_1 to E_3.

Output from pins A0-A3 may be determined by taking the modulus of the relay number and converting this number to a binary array as shown below.

DecoderNumber = RelayNumber%15

DecoderStates = bin(DecoderNumber)

where the *DecoderNumber* value indicates the active decoder output (e.g. a value of 1 would correspond to output Y1), the value of *15* corresponds to the number of outputs from each decoder, and *DecoderStates* is a list of on/off states to be sent to GPIO pins A0-A3. The *DecoderStates* list will always start with '0b,' and its components will be stored as characters. Thus, when referenced, they must be converted to integers. In place of activating a single GPIO pin, all pins must be assigned values as shown below:

GPIO.output(Relay_channel[1], int(DecoderStates[-1]))

...

GPIO.output(Relay_channel[n], int(DecoderStates[-n]))

where *n* corresponds to the number

The desired "enable" pin may be set relatively easily by applying floor division to the relay channel number as shown below:

EnableNumber = RelayNumber//15 + 1

GPIO.output(Relay_channel[n+EnableNumber], 0)

where *EnableNumber* indicates which "enable" channel is activated. For example, if relay number 16 were to be activated, *EnableNumber* would be equal to 2, activating the second decoder chip. *DecoderNumber* would be equal to 1, activating the "Y1" output of the second decoder.

Following operation of the selected pump for the desired time, the pump may be deactivated by returning the enable output to the high state (which counterintuitively disables the decoder chip) as shown below:

sleep(...)

GPIO.output(Relay_channel[n+EnableNumber],1)

Reinitialization of the other GPIO outputs is not necessary, as they will be reset prior to the activation of the next relay channel. The above code and methodology must be substituted for each step in which pumps are activated then deactivated.

Supplement D. Python3 Script for Synthesizer Operation

```
#!/usr/bin/env python
'''
Code for automated peptide synthesis
Step 1: Count the number of different amino acids used in your peptide.
Step 2: Connect GPIO pins from the Pi or decoder outputs to the relay (use
number of different peptides plus 5 GPIO pins/relay outputs). Be sure to
adjust the number of relay channels in the code as well.
Step 3: Pump 0 should pump pure DMF;
        Pump 1 should pump 20% piperidine in DMF;
        Pump 2 should pump the NMM and coupling agent;
        Pump 3 should be the draining pump for the bottom reaction vessel
        Pump 4 should be the draining pump for the top reaction vessel
Step 4: Starting from the end closest to the resin, use pump 5 for the
first
        amino acid, pump 6 for the second, etc. If an amino acid occurs
more
        than once, do not use another pump for the same amino acid.
Step 5: Run this Python file (make sure Python 3 is being used).
Step 6: Once the the file begins to run, type in the letter abbreviation
of the
        first peptide you will be using.
Step 7: The rest of the process should be done manually, but observe the
        process at all times to make sure everything runs smoothly.
'''
import RPi.GPIO as GPIO
from time import sleep
import numpy as np

'''
Initializes the GPIO pins utilized for the peptide synthesis.  These
values may be changed if different GPIO pins are used.
'''
Relay_channel = [8,10,12,16,40,22,24,26,31,33,35,37]

'''
Initializes outputs for GPIO pins.
'''
def setup():
        GPIO.setmode(GPIO.BOARD)
        GPIO.setup(Relay_channel, GPIO.OUT, initial=GPIO.HIGH)

def main():
        '''
        Drains DMF manually added to reaction vessel once 10-15 minutes
        have been allotted for swelling.
        '''
        print('DMF from resin swelling being drained.')
        GPIO.output(Relay_channel[3], GPIO.LOW)
        sleep(20)
```

```
        GPIO.output(Relay_channel[3], GPIO.HIGH)
        sleep(1)

        '''
        Validates whether peptide the user entered is valid. If it is not,
        the user is prompted to enter the name of the peptide again.
        Additional characters may be appended for use with non-natural
        amino acids
        '''
        p = 'No'
        while p != 'Yes':
                AllAmiAci =
                ['A','R','N','D','B','C','Q','E','Z','G','H','I','L','K','
                M','F','P','S','T','W','Y','V']
                print('Please write out your peptide using single letter
abbreviations.')
                print('Be sure to use all capital letters.')
                Pep = input()
                t = 'F'
                while t == 'F':
                        if set(Pep) < set(AllAmiAci):
                                t = 'T'
                                PepLen = len(Pep)
                                NumAmiAci = len(set(Pep))
                        else:
                                t = 'F'
                                print('Invalid input. Please try again.')
                                print('Please write out your peptide using
                                      single letter abbreviations.')
                                print('Be sure to use all capital
letters.')
                                Pep = input()
                print('Is ',Pep, ' the amino acid chain you would like to
                      build (type \"Yes\" or \"No\")?')
                p = input()
                if p != 'Yes':
                        print('Since this is not the peptide you wanted or
                               you do not know how to type')
                        print('\"Yes\" properly, please start the process
over.')

        '''
        Ensures user is ready to begin the peptide synthesis. If they are
        not, prompts user to begin when they are ready.
        '''
        print('Are you ready to begin your peptide synthesis (type \"Yes\"
or \"No\")?')
        r = 'F'
        while r == 'F':
                y = input()
                if y != 'Yes':
                        print('Since you are not ready or you do not know
how to type')
                        print('\"Yes\" properly, type \"Yes\" when you are
```

```
ready.')
                        r = 'F'
                else:
                        r = 'T'

        '''
        Creates an array for each amino acid in the peptide corresponding
        to the position where it first appears in the sequence. For
        example, GSGS would correspond to 1212.
        '''
        for k in range(0,PepLen-1):
                PepNum = np.linspace(1,4,PepLen)
        for i in range(0,PepLen):
                j = 0
                q = 0
                while Pep[j] != Pep[i]:
                        j = j+1
                for c in range(1,j+1):
                        for z in range(0,c):
                                if Pep[c] == Pep[z]:
                                        q = q+1
                                        break
                PepNum[i] = int(round(j-q))

        '''Adds the current amino acid to the peptide chain. '''

        for l in range(0,PepLen):
                print('Amino acid',l+1,'which is',Pep[l],'and is in in
pump',str(PepNum[l]+5)[0])
                print('will now be added. Please standby.')

                '''
                Adds 20% piperidine in DMF to the solution two times (one
                for two minutes and the second time for 6 minutes) to
                deprotect the peptide that is being added.
                '''
                print('20% piperidine in DMF being added (1st time).')
                GPIO.output(Relay_channel[1], GPIO.LOW)
                sleep(0.6)
                GPIO.output(Relay_channel[1], GPIO.HIGH)
                sleep(120)
                GPIO.output(Relay_channel[3], GPIO.LOW)
                sleep(20)
                GPIO.output(Relay_channel[3], GPIO.HIGH)
                sleep(1)
                print('20% piperidine in DMF being added (2nd time).')
                GPIO.output(Relay_channel[1], GPIO.LOW)
                sleep(0.6)
                GPIO.output(Relay_channel[1], GPIO.HIGH)
                sleep(480)
                GPIO.output(Relay_channel[3], GPIO.LOW)
                sleep(20)
                GPIO.output(Relay_channel[3], GPIO.HIGH)
                sleep(1)
```

```
'''
Washes the solution 4 times with DMF to rinse impurities
out of the reaction vessel.
'''
print('DMF washing steps now taking place.')
for k in range(0,4):
        GPIO.output(Relay_channel[0], GPIO.LOW)
        sleep(2)
        GPIO.output(Relay_channel[0], GPIO.HIGH)
        sleep(1)
        GPIO.output(Relay_channel[4], GPIO.LOW)
        sleep(20)
        GPIO.output(Relay_channel[4], GPIO.HIGH)
        sleep(1)
        GPIO.output(Relay_channel[3], GPIO.LOW)
        sleep(20)
        GPIO.output(Relay_channel[3], GPIO.HIGH)
        sleep(1)
'''
Adds a HATU solution dissolved in DMF and the amino acid
being added (also dissolved in DMF) to the reaction vessel
to add the current amino acid to the chain.
'''
print('Amino acid now being added.')
GPIO.output(Relay_channel[int(PepNum[l]+5)], GPIO.LOW)
sleep(1.25)
GPIO.output(Relay_channel[int(PepNum[l]+5)], GPIO.HIGH)
sleep(1)
GPIO.output(Relay_channel[2], GPIO.LOW)
sleep(0.625)
GPIO.output(Relay_channel[2], GPIO.HIGH)
sleep(10)
GPIO.output(Relay_channel[4], GPIO.LOW)
sleep(20)
GPIO.output(Relay_channel[4], GPIO.HIGH)
sleep(900)
GPIO.output(Relay_channel[3], GPIO.LOW)
sleep(20)
GPIO.output(Relay_channel[3], GPIO.HIGH)
sleep(1)

'''
Washes the solution 4 times with DMF to rinse impurities
out of the reaction vessel.
'''
print('DMF washing steps now taking place.')
for k in range(0,3):
        GPIO.output(Relay_channel[0], GPIO.LOW)
        sleep(2)
        GPIO.output(Relay_channel[0], GPIO.HIGH)
        sleep(1)
        GPIO.output(Relay_channel[4], GPIO.LOW)
        sleep(20)
```

```
                        GPIO.output(Relay_channel[4], GPIO.HIGH)
                        sleep(1)
                        GPIO.output(Relay_channel[3], GPIO.LOW)
                        sleep(20)
                        GPIO.output(Relay_channel[3], GPIO.HIGH)
                        sleep(1)

                '''
                Notifies the user the current amino acid is done being
                added and asks if the user is ready to add the next amino
                acid in the sequence (if applicable). If the sequence
                completed, the code terminates.
                '''
                print('This amino acid step is complete.')
                if l != PepLen-1:
                        print('Are you ready to add your next amino acid
(type \"Yes\" or \"No\")?')
                        u = 'F'
                        while u == 'F':
                                p = input()
                                if p != 'Yes':
                                        print('Since you are not ready or
                                                you do not know how to
                                                type')
                                        print('\"Yes\" properly, type
\"Yes\" when you are ready.')
                                        u = 'F'
                                else:
                                        u = 'T'
                                        print('Moving on to the next amino
acid.')
                else:
                        print('Your peptide is done being made.')
                        print('Be sure to dispose of everything properly
and to clean all equipment.')

'''
Default settings if the code is terminated anytime during its execution.
'''
def destroy():
        GPIO.output(Relay_channel, GPIO.LOW)
        GPIO.cleanup()

if __name__ == '__main__':
        setup()
        try:
                main()
        except KeyboardInterrupt:
                destroy()
```

Supplement E. Reagent Cost Estimate

Table D. SPPS Reagent Pricing

Reagent	Initially used	Amt per step	Cost/mL or g	Cost/Step, $	Initial Cost
DMF	100mL	12.45mL	0.0435	0.541575	0.435
Piperidine	0mL	0.3mL	0.1085	0.03255	0
NMM	0mL	0.026875mL	0.0908	0.00244025	0
HATU	0g	0.046875g	7.2	0.3375	0
Amino acid	0g	0.0405g	2.5	0.10125	0
Resin	0.05g	0g	4	0	0.2
			**Startup Cost, $**	0.635	
			**Cost/Step, $**	1.01531525	

Supplement F. Testing HA-tag peptide

Tests performed with HA-tags and anti-HA-tag monoclonal antibodies were less conclusive (Fig C). While HA-tagged beads, on average, were slightly brighter than control beads, fragmented resin beads exhibited significantly brighter fluorescence. This may result from weaker HA-tag antibody affinity relative to streptavidin affinity and polystyrene-protein affinity.

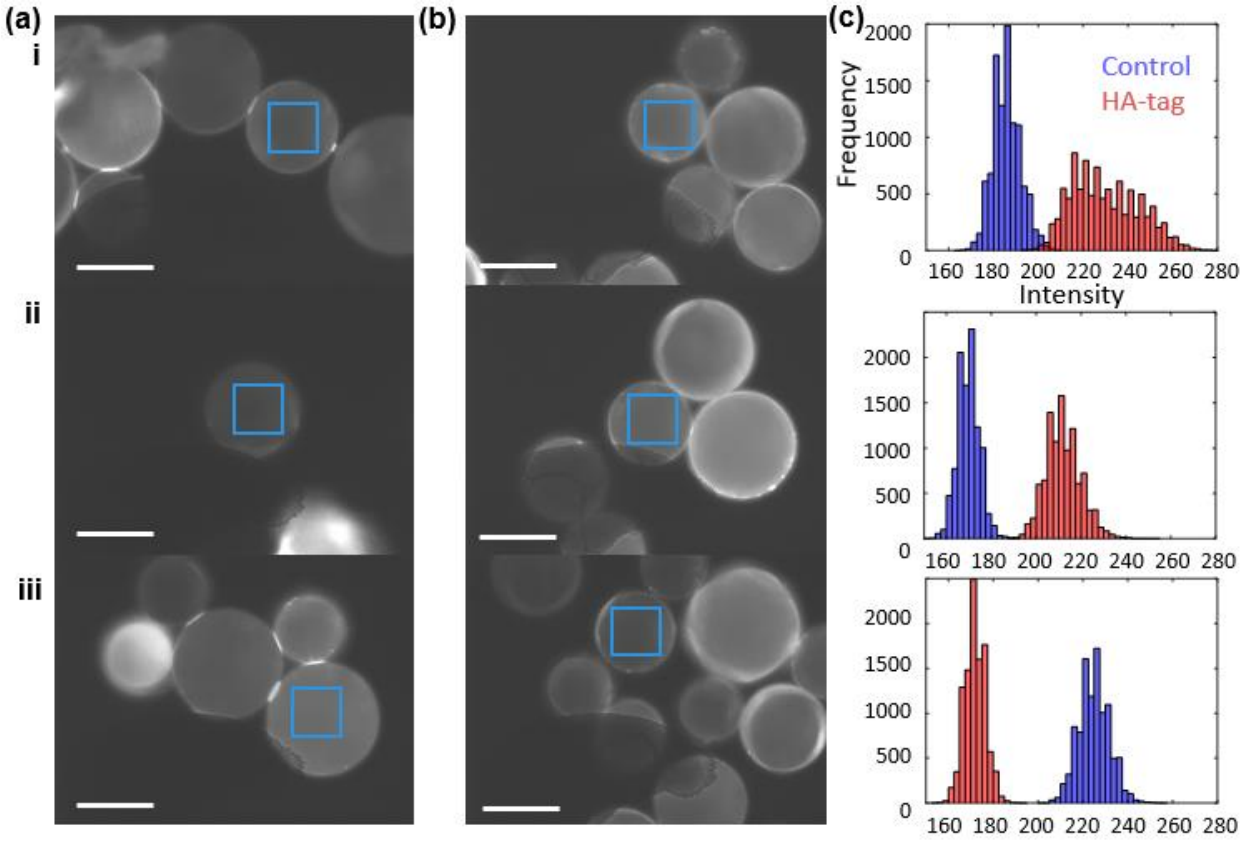

Fig C. Fluorescent microscope image of aminomethyl styrene resin functionalized with **(a)** control $(GS)_4$ sequence and **(b)** strep-tag after incubated with DyLight 488-anti-HA tag. **(c)** Histogram comparison of pixel intensities collected from regions of interest (solid blue boxes in a-b). **(i-iii)** Replicates. Scale bars are 50 µm.
